# Methodologies to Identify Metabolic Pathway Differences Between Emaciated and Moderately Conditioned Horses: A Review of Multiple Gene Expression Techniques

**DOI:** 10.3390/ani15202933

**Published:** 2025-10-10

**Authors:** Madeline M. P. Austin, Jennie L. Z. Ivey, Elizabeth A. Shepherd, Phillip R. Myer

**Affiliations:** Department of Animal Science, University of Tennessee, 2506 River Drive, Knoxville, TN 37996, USA

**Keywords:** starvation, metabolic alteration, gene expression, RNA-sequencing, RT-qPCR

## Abstract

Starvation in horses presents critical welfare, economic, and management challenges with underlying molecular mechanisms of metabolic modification and recovery left poorly defined. Prolonged caloric deprivation induces significant systemic shifts in carbohydrate, protein, and lipid metabolism, reflected in coordinated changes in tissue-specific gene expression. This review synthesizes current knowledge on equine metabolic responses to starvation, emphasizing pathways found through RNA sequencing (RNA-seq) and real-time quantitative polymerase chain reaction (RT-qPCR) studies. Molecular investigations using RNA-seq and RT-qPCR have provided insight into transcriptional reprogramming during starvation and subsequent refeeding. Shifts in gene expression reflect the metabolic transition from carbohydrate dependence to lipid use, suppression of anabolic signaling, and activation of proteolytic pathways. However, interpretation of these data requires caution, as factors such as post-mortem interval, tissue handling, and euthanasia methods particularly the use of sodium barbiturates can influence transcript stability and abundance, potentially confounding results. The literature shows that starvation-induced molecular changes are not uniform across tissues, with skeletal muscle, liver, and adipose tissue showing distinct transcriptional signatures and variable recovery patterns during refeeding. Cross-species comparisons with hibernation, caloric restriction, and cachexia models provide context for understanding these changes, though equine-specific studies remain limited. Identified gaps include the scarcity of longitudinal data, inconsistent tissue sampling protocols, and lack of standardized reference genes for transcriptomic analyses in horses. Addressing these limitations will improve the accuracy of molecular evaluations and enhance our ability to predict recovery trajectories. A more comprehensive understanding of systemic and tissue-specific responses to starvation will inform evidence-based rehabilitation strategies, reduce the risk of refeeding syndrome, and improve survival and welfare outcomes for affected horses.

## Simple Summary

Starvation in horses results in profound metabolic adjustments, including the breakdown of fat and muscle to sustain energy demands when restricted feed intake occurs. Molecular techniques, such as RNA sequencing (RNA-seq) and real-time quantitative polymerase chain reaction (RT-qPCR), can be used to examine physiological changes and to analyze gene expression in tissues including muscle, liver, and adipose tissue. Such research offers critical insight into the mechanisms of metabolic alterations and the processes involved during refeeding. Continued investigation using these advanced methodologies is necessary to inform evidence-based rehabilitation protocols that enhance recovery outcomes and improve the welfare of malnourished equines.

## 1. Introduction

The increasing prevalence of emaciated horses in the United States presents significant welfare, economic, and ethical challenges. Approximately 130,000 horses are classified annually as unwanted, with many suffering from neglect, starvation, and eventual death [1]. Increased economic strain, population, and processing regulation changes have impacted the equine industry and has raised ethical concerns surrounding proper care and management of these animals [2]. Addressing the challenges of refeeding starved horses is critical, as recovery is complicated by insufficient understanding of the metabolic changes associated with starvation and subsequent rehabilitation [3]. Body condition scoring (BCS) remains the standard method of evaluating equine health; however, BCS provides a limited perspective on complex metabolic disruptions occurring during periods of starvation [4].

Neglected horses require intensive medical care, rehabilitation, and refeeding programs, which involve substantial costs. Veterinary care, including diagnostics, nutritional supplements, and emergency treatments can strain limited resources, while the cost of humane euthanasia for horses deemed beyond recovery adds an additional expense [5,6]. Rehoming starved horses is fraught with difficulty, as underconditioned animals may experience long-term health complications even after rehabilitation [3]. The lack of enforceable legislation and standardized care practices leaves many horses vulnerable to starvation and mistreatment [7]. Owners may lack sufficient knowledge or financial resources to provide adequate care, leading to deteriorating health conditions and poor quality of life for the animals [8]. The economic implications extend to the equine industry as overpopulation contributes to the saturation of horse markets, decreasing the value of horses and leaving animals without homes or proper care. Rescue organizations and sanctuaries are often overwhelmed with the sheer volume of horses in need, further amplifying financial and logistical pressures on the industry [9]. The growing population and lack of resources to match underscores the urgent need for industry-wide solutions addressing both the root causes of neglect, and the challenges associated with caring for emaciated horses. 

Despite its critical importance, the understanding of metabolic changes in starved horses remains limited. Starvation triggers widespread alterations in metabolic pathways, including disruptions in glucose metabolism, oxidative phosphorylation, and lipid oxidation [1,10]. While research in humans and other mammalian species has provided insights into these mechanisms, the physiological responses in horses are less well-characterized [11,12,13,14]. Equine metabolism is uniquely adapted to an herbivorous diet and high reliance on hindgut fermentation, making their responses to starvation distinct from those of other species and requiring an emphasis on microbial fermentation and systemic stress in regard to starvation and recovery [15]. Preliminary studies suggest nutrient metabolism in starved horses is severely impaired, with potential long-term consequences for recovery and energy homeostasis [1,16]. These gaps in knowledge hinder the development of evidence-based refeeding protocols and emphasize the need for further research on tissue-specific gene expression and metabolic alteration in starved equids [3].

Mediation of starvation-associated metabolic changes occurs in part by the transcriptional regulation of key genes involved in energy balance. GLUT4 (glucose transporter type 4), PDK4 (pyruvate dehydrogenase kinase 4), PFKM (phosphofructokinase muscle), CPT1B (carnitine palmitoyltransferase 1B), and MSTN (myostatin) are among the most extensively investigated genes in equine research, frequently identified as central regulators of metabolic function. Their repeated implication in studies regarding starvation, cachexia, and hibernation further emphasizes their relevance, making them particularly well suited for focused examination of response to chronic nutrient deprivation. In the context of gene function, GLUT4 encodes a glucose transporter critical for skeletal muscle glucose uptake; its downregulation during nutrient deprivation reflects reduced insulin sensitivity and thus diminished carbohydrate utilization [17]. PDK4 inhibits pyruvate dehydrogenase activity, thereby limiting glucose oxidation and redirecting substrates toward lipid metabolism, a hallmark response to negative energy balance [13]. PFKM, which encodes the muscle isoform of phosphofructokinase, is central to the glycolytic flux and may be suppressed under starvation to conserve glucose for vital tissues [18]. CPT1B facilitates the transport of long-chain fatty acids into mitochondria for β-oxidation, a process upregulated to meet energy demands during prolonged fasting [15]. MSTN, a regulator of muscle growth, is often elevated in catabolic states, promoting protein degradation and contributing to muscle wasting seen in emaciated horses [11,16]. Together, these genes highlight the shift from carbohydrate to lipid and protein catabolism during starvation, underscoring the importance of gene expression tools, such as Real Time quantitative Polymerase Chain Reaction (RT-qPCR) and RNA-Sequencing (RNA-seq), to better understand equine responses to long-term nutritional deprivation.

Refeeding starved horses is a complex and high-risk process, often filled with challenges such as refeeding syndrome, a potentially fatal condition caused by the rapid introduction of nutrients into the system of a chronically malnourished animal [19]. Due to reduced insulin and increased glucagon secretion during prolonged starvation, rapid introduction of carbohydrates leads to a sudden shift from endogenous energy substrates to insulin-driven utilization of exogenous glucose for energy. This response leads to a shift of glucose into the cells and thus drives the migration of electrolytes into the cell [20]. The combined effects of a depleted internal store of electrolytes with rapid intracellular migration of these electrolytes induces hypokalemia, hypophosphatemia, and hypomagnesemia [20]. These complications can lead to heart failure, respiratory failure, anemia, hemolysis, and immune suppression, and eventually death [21]. Additionally, the optimal composition and schedule for reintroducing nutrients to severely underweight horses remains poorly defined [22]. Research in other species suggests that gradual refeeding is critical for avoiding complications, yet there is little consensus on the best practices for horses [23]. Thus, there is an urgent need for detailed investigations into the metabolic responses of starved horses, including the role of tissue-specific pathways in recovery. Advancements in diagnostic tools, such as RNA- seq, could offer valuable insights into the molecular processes underlying starvation and refeeding, paving the way for more effective and safe rehabilitation practices [24].

## 2. Methodology

### 2.1. Search Strategy

A targeted literature search was conducted to identify peer-reviewed journal articles relevant to the metabolic acclimatization to starvation in horses and other mammals. Primary search tools included Google Scholar, PubMed, and MDPI supplemented with manual reference list checks from relevant articles. Search terms included combinations of keywords such as starvation, cachexia, hibernation, negative energy balance, gene expression, metabolic alteration, and mammals. Additional searches were conducted using the names of specific genes of interest (e.g., GLUT4, PDK4, PFKM, MSTN, and CPT1B) combined with terms such as starvation, fasting, nutritional deprivation, or energy metabolism. While equine-based literature remained the primary focus, review of studies in other mammalian species occurred to provide context and to bridge knowledge gaps where equine-specific research was minimal. Due to the nature of separate searches with specific criteria, relative content may have been excluded unintentionally; however, not to impact the integrity of the sources utilized in the review.

### 2.2. Inclusion and Exclusion Criteria

No formal date restrictions were applied; however, recent publications were specifically sought out to reflect current methodological approaches such as RNA-seq and RT-qPCR. Studies were excluded if they were not peer-reviewed, were deficient in empirical findings, or focused solely on non-mammalian species without relevant translational insight, such as opinion pieces or narrative reviews. Articles were included if they directly involved horses, particularly those examining physiology, metabolism, or gene expression. Specific attention was given to studies if they investigated systemic or tissue-specific responses to altered energy balance, including changes in skeletal muscle, liver, and adipose tissue. Highly relevant research included that which employed transcriptomic or molecular approaches such as RNA sequencing, microarrays, or real-time quantitative polymerase chain reaction to measure gene expression changes. Comparative studies in other mammalian species examining physiological states with metabolic parallels to equine starvation, such as hibernation, cachexia, or prolonged exercise were included. This approach ensured that the literature reviewed captured both equine-specific evidence and comparative mammalian insights, supporting a comprehensive evaluation of metabolic and transcriptional responses to starvation. 

### 2.3. Quality Assessment

The quality of the studies reviewed varies, with some providing robust experimental designs and others having limitations with small sample sizes or lack of longitudinal data. For example, Main et al. conducted a comprehensive metabolomic analysis during the refeeding of starved horses, offering valuable insights into recovery processes [1]. In contrast, older studies, such as Witham & Stull, while foundational, lacked the advanced molecular tools available today [15]. Recent meta-analyses and systematic reviews in the field, such as those examining global trends in starvation recovery across species, have further highlighted the importance of standardized methodologies and large-scale datasets to enhance reproducibility and comparability. By synthesizing data from multiple studies, these reviews help identify additional areas of necessary research and provide a clearer picture of effective interventions.

## 3. Summary of Key Findings

### 3.1. Discussion of Major Themes

The empirical literature on metabolic gene expression in horses provides critical insights into the impact of starvation on equine physiology and the potential pathways for recovery [25]. Hibernation and cachexia can serve as comparative frameworks for understanding starvation mechanisms in horses regarding the role of metabolic genes in adaptive and maladaptive responses to prolonged nutritional deprivation. Studies have illuminated the seasonal and adaptive changes in gene expression in hibernating species as well as upregulation of genes related to fatty acid metabolism during cachexia [26,27,28]. Additional studies in rodents and primates shed light on the conservation of metabolic pathways across species, highlighting unique metabolic adjustments in response to nutrient scarcity, further enriching our understanding of equine responses by providing broader biological contexts [27]. Seasonal and adaptive changes in gene expression, particularly in hibernating species, offering a comparative framework for understanding similar mechanisms during starvation in horses, especially in context of utilizing gene expression to find metabolic pathway changes [29,30].

The interplay between starvation and inflammation plays a role in both gene expression and the process of emaciation over time. Elevated levels of inflammatory cytokines during nutrient deprivation have been found in livestock, which parallels findings in horses, exhibiting a final attempt to increase immune cell chemotaxis and decrease systemic inflammation associated with the stress of emaciation [11,31]. Laboratory models, such as those conducted on rodents, also provide evidence of the critical role inflammation plays in mediating recovery outcomes, further supporting the need for targeted anti-inflammatory interventions during the refeeding process in equines [32]. Furthermore, inflammatory responses to starvation can affect gene expression outcomes due to expression specifically related to inflammation rather than the underlying condition of starvation or similar chronic illness [32]. To accurately account for genomic adjustment to starvation, it is important to understand the standalone impact of inflammation on gene expressions.

The adaptive role of metabolically related genes in the context of starvation and chronic undernutrition in horses stands out as another major theme at play. Prolonged starvation in horses induces systemic shifts in metabolic priorities regulated by key genes. Carbohydrate metabolism is suppressed in peripheral tissues, with decreased expression of SLC2A4 (solute carrier family 2 member 4) and HK2 (hexokinase 2), while genes such as PDK4, PCK1 (phosphoenolpyruvate carboxykinase 1), and G6PC (glucose-6-phosphatase) are upregulated to promote gluconeogenesis and limit glucose oxidation, conserving glucose for obligate tissues [33,34,35,36,37]. Muscle catabolism is driven by increased expression of FBXO32 (f-box only protein 32, atrogin-1) and TRIM63 (muscle RING finger 1), providing amino acids for gluconeogenesis but causing significant muscle loss [38]. Concurrently, lipid metabolism genes including CPT1, PPARGC1A (peroxisome proliferator activated receptor gamma coactivator 1-alpha), and FABP4 (fatty acid binding protein 4) are upregulated to enhance fatty acid mobilization and B-oxidation, compensating for reduced carbohydrate availability [39,40,41]. These coordinated transcriptional changes illustrate the metabolic transition from glucose reliance to protein and fat utilization, highlighting gene expression as a marker of nutritional stress and a potential tool for evaluating equine body condition and guiding rehabilitation strategies.

Across transcriptomic studies involving post-mortem tissue is the potential confounding impact of euthanasia method—particularly sodium barbiturate overdose—on gene expression. While barbiturates such as sodium pentobarbital are widely used for humane euthanasia in horses and other mammals, they have been shown to induce rapid transcriptional and metabolic changes prior to and during death [42,43,44]. In addition to the primary action of sodium barbiturates on GABAα receptors, there is also modulation of inflammatory pathways by suppressing NF-kβ signaling, consequently downregulating pro-inflammatory cytokines which can distort tissue transcriptomal profiles. In parallel, barbiturates depress mitochondrial oxidative phosphorylation and reduce ATP production, leading to a broad suppression of metabolic gene expression and altered representation of pathways related to glycolysis and biosynthesis [45]. Additionally at the tissue level, barbiturate euthanasia introduces structural artifacts, including vacuolization and abnormal cellular morphology, complicating both histological and molecular studies [46]. The impact extends to RNA integrity, as barbiturate-based euthanasia is associated with reduced RNA yield and compromised quality relative to physical or alternative chemical methods, largely due to ischemia-induced degradation processes [47]. Such effects are especially concerning in studies comparing physiologically distinct groups, like emaciated and moderately conditioned horses, where minor differences in gene expression may be obscured or amplified by the euthanasia protocol itself. Standardizing euthanasia procedures, minimizing time to tissue collection, and recognizing the molecular effects of barbiturates are essential to ensuring valid interpretation of RNA-seq and RT-qPCR data in post-mortem equine research. 

### 3.2. Methodological Approaches and Relative Limitations

Investigation of metabolic changes in emaciation horses has incurred the application of different methodologies, such as observational analyses and advanced molecular techniques. Among these, RT-qPCR remains widely used for assessing gene expression due to its sensitivity, specificity, cost effectiveness, and ease of use [48,49]. An essential first step in accurate RT-qPCR analysis is the selection of stable reference genes. For instance, Bogaert et al. identified a reliable set of reference genes in normal equine skin and equine sarcoids, emphasizing that selecting appropriate housekeeping genes is vital for normalizing target gene expression data [49]. Reliable reference gene identification is echoed in studies on caloric restriction in mice and hibernating species, underscoring that reference gene selection can vary widely among tissues, species, and physiological states [48,50,51]. However, limitations remain due the reliance on pre-selected genes, the potential for normalization errors, and restricted ability to capture broad transcriptomic changes or rare pathways [24,52].

RNA-seq has emerged as an alternative that provides a broader, unbiased view of transcriptomic activity, enabling the identification of novel gene pathways and comprehensive expression patterns [18,53]. RNA-Seq has become an indispensable tool in metabolic research due to its ability to provide comprehensive insights into gene expression profiles [24]. Unlike traditional methods such as microarrays, RNA-Seq offers a broader dynamic range and higher sensitivity, enabling the detection of both abundant and rare transcripts. This technology allows for the identification of novel transcripts, alternative splicing events, and non-coding RNAs, which are crucial for understanding complex metabolic pathways [18]. A review by Wang et al. highlights that RNA-Seq has transformed transcriptomics by allowing analysis of expressed genes with exon-level resolution across various tissues and species, without prior knowledge of the transcriptome [51]. In equine studies, RNA-Seq has been effectively utilized to explore the molecular responses to physiological challenges. Capomaccio et al. applied RNA-Seq to analyze the exercise-induced stress transcriptome in endurance horses, uncovering specific gene expression changes associated with metabolic adaptations during prolonged physical activity [17]. These findings demonstrate the utility of RNA-Seq in identifying key metabolic pathways and regulatory mechanisms in horses.

RNA-Seq has also been employed to study the effects of dietary interventions on gene expression in various tissues. A study comparing microbiota characterization in horses fed a high fiber or high starch diet found that horses being fed a high fiber diet had more variation in their gut microbiota than those being fed a high starch diet [54]. Significance found in gene expression based on dietary changes indicates a role played by metabolically related genes throughout the digestive tract to maintain homeostasis. Additionally, because age, breed, and feeding management affects gut microbiota, starvation may have a significant impact on the ability of the gut microbiota to remain in symbiosis, thus creating differences in gene expression and overall digestive homeostasis. This has been shown in other mammalian species with the degradation of microbiome diversity and bacterial species during starvation, but after refeeding, the gut microbiome is restored and symbiosis continued to support digestive homeostasis [25]. Despite these advantages RNA-seq requires advanced bioinformatics expertise, substantial computational resources, and often produces large datasets that can complicate interpretation [55]. Combining RT-qPCR and RNA-seq in integrative approaches allows researchers to balance targeted validation with comprehensive discovery, generating robust, multidimensional data and improving the reliability and scope of equine gene expression research.

Gene expression differs significantly between live and deceased animals due to the cessation of active biological processes after death and the onset of post-mortem molecular changes [56]. In living animals, gene expression is a dynamic process regulated by environmental cues, metabolic needs, and homeostatic mechanisms. Active transcription ensures that proteins and enzymes necessary for physiological functions are synthesized and maintained [57]. This real-time adaptability allows researchers to study gene expression as a reflection of an organism’s current physiological state, enabling insights into metabolic activity, immune responses, and other biological processes. In deceased animals, however, gene expression changes drastically as cells lose access to oxygen and energy sources, leading to the cessation of active transcription and translation, degrading RNA quality and increasing instability [58]. Post-mortem, genes involved in stress responses, apoptosis, or immune pathways, may exhibit transient expression due to the body’s final attempts to maintain cellular integrity [58]. Studies have documented the “thanatotranscriptome,” which refers to the unique transcriptional activity observed after death [59]. By understanding these differences, researchers can better design studies to analyze gene expression in the appropriate context, whether it involves exploring active biological processes in living animals or investigating molecular changes in deceased specimens for forensic or pathological applications [60].

### 3.3. Euthanasia Method and Gene Expression

An emerging concern in transcriptomic studies using post-mortem tissue is the potential influence of euthanasia methods on gene expression, particularly with the use of sodium barbiturates. Sodium pentobarbital, a central nervous system depressant commonly employed in both laboratory and veterinary settings, induces rapid unconsciousness and cessation of respiration through potentiation of GABA-A receptors and widespread inhibition of excitatory neural activity [45]. While having a well-established humane application, studies have demonstrated that barbiturate-based euthanasia can produce downstream molecular effects that complicate the interpretation of gene expression data. In murine models, it was found that both anesthesia and euthanasia methods significantly altered the metabolomic profiles of tissues, including the liver and muscle, suggesting that transcriptional and metabolic changes can be rapidly induced even before death is complete [42]. Similarly, Mohamed et al. reported that the dose of sodium pentobarbital administered prior to exsanguination affected not only biochemical and histological features but also gene expression outcomes in rat tissue, further indicating that euthanasia-induced stress responses may confound molecular analyses [43].

Other mammalian systems mirror these findings. Documentation exists with tissue artifacts induced by barbiturate euthanasia in nonhuman primates, emphasizing histological distortions and localized tissue disruption that could interfere with downstream RNA extraction and analysis [46]. In more recent studies, it was demonstrated that barbiturate-derived compounds inhibited the expression of inflammation-associated genes, such as MMP9, through a cGMP-dependent signaling cascade, illustrating that barbiturates may directly modulate transcription in certain tissues [44]. Other observations exist stating that barbiturate and other anesthetic agents altered inflammatory signaling in cultured epithelial and hepatic cell lines, suggesting that even short-term exposure to these drugs can shift transcriptional dynamics [61]. A comparative study by Bhaskaran et al. on porcine tissues revealed that both euthanasia method and post-mortem handling significantly influenced RNA yield and quality, underscoring the importance of immediate tissue processing and consistent protocols in transcriptomic studies [47].

In the context of equine research, where euthanasia is often necessary for tissue collection from severely emaciated or moribund individuals, these considerations are especially pertinent. Although sodium pentobarbital remains the most widely used euthanasia agent in horses due to its humane profile and widespread acceptance, its potential to induce gene expression changes prior to death poses a methodological challenge [62]. Variability in dosing, the use of adjunctive anesthetics, and time intervals between euthanasia and tissue sampling can all influence the transcriptome in ways that mimic or obscure true physiological differences related to body condition or disease state. As such, studies utilizing tissue samples for gene expression must account for euthanasia effects in their experimental design and data interpretation. Employing rapid tissue collection protocols, consistent dosing regimens, and appropriate controls may help mitigate the confounding impact of barbiturate euthanasia on gene expression profiles, improving the reliability and biological relevance of RNA-seq and RT-qPCR findings in equine metabolic research.

To avoid these confounding factors, propositions of alternative euthanasia methods, such as humane gunshot or intrathecal lidocaine, exist. These methods are suggested for cases where preserving the integrity of molecular data is critical, such as in studies examining the effects of starvation, metabolism, or gene regulation [62]. The negative effects of SB euthanasia could confound results in studies utilizing gene expression to quantify metabolic pathway changes during starvation or other conditions where precise molecular measurements are required [47]. While widely used in the equine industry due to their efficacy, researchers must consider the potential impact of sodium barbiturates on post-mortem gene expression and explore alternative methods when studying molecular or biochemical processes.

### 3.4. Controversies in the Literature

Vast differences in study design, including live versus dead sampling, sample size, tissue collection timing, and the challenges of translating findings from model organisms and alternative species compared to horses can lead to discrepancies between studies [63]. Within equine-based studies utilizing RT-qPCR and RNA-seq, many tissues are collected from both live and euthanized equids including cardiac muscle, liver, gastrointestinal tract, and most predominantly, skeletal muscle [64,65,66,67,68,69]. Additionally, sample sizes range from six horses to over one-hundred horses and there are many breeds represented across the literature, possibly contributing to differences in overall metabolic function for both RT-qPCR and RNA-seq studies, compounding the concern for inconsistencies [70,71,72,73,74,75,76]. Sample sizes in non-equid mammals also have a wide range, being very species dependent, contributing to varied findings across multiple species utilizing both RNA-seq and RT-qPCR [25,27,56,57,77,78,79]. While hibernating mammals offer a valuable comparative model for understanding starvation-induced gene expression, direct application of these findings to equines is complicated by species-specific metabolic differences [29,80]. These methodological and species-specific differences underscore the need for cautious interpretation of gene expression data and highlight the importance of standardized approaches in equine-focused starvation research.

### 3.5. Patterns and Inconsistencies

Patterns across studies indicate a consensus on the importance of metabolic changes during starvation, but inconsistencies remain in the interpretation of these changes. While the protective role of autophagy during nutrient deprivation has been noted, it has also been suggested that excessive activation of these pathways can lead to detrimental effects, such as tissue damage [11,14]. Studies in human medicine, such as those examining starvation in malnourished patients, similarly show a dual role of autophagy, where its regulation is critical to preventing organ damage [81]. Additionally, research in veterinary medicine has demonstrated varied responses to nutrient deprivation across species, emphasizing the need for species-specific protocols [82]. These cross-disciplinary examples highlight the complexity and variability of metabolic responses, underlining the importance of tailored approaches in equine studies.

Assessing health in emaciated horses presents unique challenges, as commonly employed measures may not fully capture underlying physiological status. Among these, body condition score (BCS) is widely used in both research and clinical contexts, yet its reliability as a predictor of metabolic and health outcomes remains contested [3]. While BCS is often associated with health outcomes, no correlation between BCS and specific health markers has been identified [4,83]. Furthermore, metabolic variation due to genetic predisposition for muscle type percentages exists among horse breeds, even with light breeds such as Thoroughbreds, Arabians, and Quarter Horses exhibiting distinct physiological differences that influence energy metabolism, exercise capacity, and stress responses [64,70,71,72,75]. For example, transcriptome analyses in Thoroughbreds have revealed extensive gene expression changes in response to exercise, particularly in pathways linked to energy metabolism, muscle contraction, and stress responses, emphasizing the breed’s highly specialized physiological adaptations for endurance and speed [84]. In contrast, studies of American Quarter Horses have identified genomic signatures of selection associated with performance traits such as sprinting ability, muscle fiber composition, and energy utilization [70]. Together, these studies suggest that metabolic and genetic modifications are not uniform across breeds but instead reflect distinct selective pressures. However, there remains a lack of comprehensive comparisons across a larger range of breeds leaving critical gaps in understanding how breed-specific genetics and physiology influence stress responses and overall equine health.

## 4. Metabolic Alteration to Starvation and Related Gene Expression in Equine Tissues

Beyond the technical aspects of gene quantification, specific metabolic pathways have been implicated starvation adjustment and subsequent refeeding in horses [1]. During carbohydrate restriction, specific genes controlling glucose uptake and fatty acid metabolism play central roles in maintaining energy homeostasis [81]. For example, GLUT4 may be particularly relevant in starved horses due to its role in signaling insulin secretion in the presence of blood glucose, which is decreased in periods of nutrient deprivation, therefore reducing overall carbohydrate metabolism, where glucose availability and insulin sensitivity can be significantly altered [85]. Ribosomal protein genes, such as RPL32, have also been investigated for their role in gene expression regulation, underscoring their potential to serve not only as reference genes but also as indicators of cellular protein synthesis capacity during times of nutritional stress [86]. Genes involved in energy substrate utilization often undergo marked shifts in expression in response to starvation. PDK4 is known to be regulated by glucocorticoids and insulin [87]. In starved or malnourished horses, upregulation of PDK4 could limit glucose oxidation by inhibiting pyruvate dehydrogenase, thereby conserving glucose for tissues that are critically dependent on it (e.g., the nervous system). CPT1A similarly facilitates entry of long-chain fatty acids into mitochondria for β-oxidation [39]. During prolonged energy deficits, increased CPT1A expression could reflect a metabolic switch favoring fatty acid utilization over carbohydrate oxidation, a phenomenon likely critical to the survival of severely malnourished horses [19].

More comprehensive characterization of metabolic trajectory of starved horses might occur by integrating gene expression analyses with clinical assessments, particularly during the delicate refeeding period. Tracking expressions of genes such as PDK4 and CPT1A, alongside other metabolic regulators, may offer insights into whether a horse is successfully adapting to nutritional rehabilitation or experiencing complications. In parallel, the use of carefully validated reference genes is crucial for ensuring the accuracy and reliability of these molecular measurements. Collectively, these advances highlight the growing importance of molecular and genetic tools in understanding and managing malnutrition and starvation in equine populations. 

### 4.1. Carbohydrate Pathway(s) and Relevant Genes

Carbohydrate metabolism is a vital component of energy regulation, particularly in large herbivores such as horses, where muscle glucose uptake and utilization are tightly regulated by hormonal and transcriptional signals. Investigation of carbohydrate related genes in horses has occurred, each highlighting a distinct regulatory point within glycolysis, glucose transport, or substrate utilization, providing a glimpse of genetic contributors to equine carbohydrate metabolism (Table 1). Among these glucose transporters, GLUT4 (SLC2A4) plays a pivotal role in insulin-mediated glucose uptake, particularly in skeletal muscle and adipose tissue [33]. In energy-deprived and insulin-sensitive states, GLUT4 expression is typically downregulated, limiting glucose entry into cells and contributing to impaired insulin sensitivity. This sensitivity leads GLUT4 to translocate to the cell membrane to facilitate glucose entry; however, its expression and translocation are impaired in insulin-resistant conditions, leading to diminished glucose uptake [88]. Diminished glucose uptake drives a starved horse further into gluconeogenesis, contributing to loss of mass in the muscle [89]. HK2 catalyzes phosphorylation of glucose to glucose-6-phosphate, the first step in glycolysis. Expression of HK2 reflects the metabolic state of the cell, typically increasing in anabolic conditions and decreasing during catabolic stress [34,90]. Expression of HK2 is regulated by the Akt/mTOR pathway, indicating a connection between energy status and glycolytic flux due to decreased glucose conversion to pyruvate [91]. PDK4 expression increases during fasting and other conditions associated with a metabolic shift from glucose to fatty acids as energy sources [35]. The adaptive response of PDK4 helps conserve glucose for tissues with obligatory glucose requirements. PFKM catalyzes the committed step of glycolysis, converting fructose-6-phosphate to fructose-1,6-bisphosphate, a rate-limiting step that commits glucose to energy production through the glycolytic pathway [92]. Activity of PFKM is strongly regulated by allosteric effectors, including fructose-2,6-bisphosphate, ATP, and citrate, allowing fine-tuned control of glycolytic flux in response to cellular energy status [92]. In prolonged energy deficiency, downregulation of PFKM activity can slow glycolysis, conserving circulating glucose for tissues with strict glucose requirements, such as the brain and red blood cells, while shifting skeletal muscle metabolism toward increased fatty acid oxidation [93]. The interplay between GLUT4, HK2, PDK4, and PFKM demonstrates a tightly coordinated network governing carbohydrate metabolism, where alterations in expression can redirect energy utilization, diminish glucose availability, and exacerbate systemic metabolic imbalances under conditions of chronic nutrient deficiency.

### 4.2. Proteins

Amino acid metabolically related genes are highly responsive to nutritional status, particularly in the context of muscle protein synthesis and degradation. Few amino-acid related genes have been studied in horses, with investigations highlighting distinct roles in protein turnover, muscle growth and atrophy, and catabolic regulation, providing molecular factors associated with equine amino acid metabolic pathways (Table 2). For example, MSTN, a member of the transforming growth factor-β superfamily, acts as a negative regulator of muscle growth. Mutations or polymorphisms in the MSTN gene have been associated with variations in muscle mass and performance traits in horses. Elevated MSTN expressions are linked to muscle wasting and sarcopenia, commonly observed in undernourished or emaciated animals [66]. Reduced expression or functional mutations can lead to increased muscle mass [71]. In contrast, IGF1 (insulin-like growth factor 1) promotes anabolic processes including protein synthesis, muscle regeneration, and satellite cell activation. Nutritional status significantly influences IGF1 levels, with undernutrition leading to decreased IGF1 expression and contributing to reduced muscle protein synthesis and growth retardation [96,97]. Further, genes such as FBXO32 and TRIM63 are central to the ubiquitin–proteasome pathway, mediating muscle protein degradation. Both are commonly upregulated during muscle atrophy and serve as molecular markers of catabolic activity in skeletal muscle [38]. Collectively, these genes highlight the shift from protein anabolism in moderately conditioned horses to catabolism and proteolysis in emaciated conditions.

### 4.3. Lipids

Lipid metabolism genes are vital in maintaining energy homeostasis, especially when carbohydrate availability is limited. In horses, prolonged energy deficit leads to increased lipolysis and fatty acid oxidation, processes regulated by key genes (Table 3). CPT1B, located on the mitochondrial membrane, is a rate-limiting enzyme in the transport of long-chain fatty acids into mitochondria for β-oxidation. Its expression is upregulated in conditions of increased lipid utilization, such as fasting and emaciation [39]. ACADM (acyl-CoA dehydrogenase, medium chain) and ACADL (long-chain acyl-CoA dehydrogenase) catalyze key steps in mitochondrial fatty acid β-oxidation and are critical for sustained lipid-based energy production [40,41]. The transcriptional coactivator PPARGC1A regulates mitochondrial biogenesis and oxidative metabolism, and is typically upregulated in response to exercise, cold exposure, and caloric restriction, promoting efficient lipid utilization and enhancing oxidative capacity [100]. FABP4, highly expressed in adipose tissue, facilitates the intracellular transport of fatty acids and is often elevated when fat stores are mobilized [101]. Furthermore, LPL (lipoprotein lipase) hydrolyzes triglycerides in lipoproteins to free fatty acids and glycerol, playing a central role in delivering lipid fuel to energy-demanding tissues [102]. Together, lipid associated genes underscore the metabolic alteration toward lipid oxidation in horses with reduced adipose and glycogen stores. 

## 5. Gaps Within the Current Available Literature on Nutrition, Metabolic, and Starvation Research in Equids

The metabolic challenges faced by starved horses underscore the necessity for a deeper understanding of their physiological responses to prolonged nutritional deprivation. Horses subjected to starvation experience alterations in critical metabolic pathways, particularly those involving glucose metabolism, lipid oxidation, and mitochondrial functions [1,10,11]. Despite these insights, research into the specific molecular responses in horses remains limited compared to studies in other species, highlighting a critical gap in equine metabolic research [13,14].

RNA-seq has emerged as a transformative tool for metabolomic research, offering unparalleled sensitivity in detecting genome-wide transcriptomic changes [18,24,51]. Unlike RT-qPCR, which is limited by its targeted approach, RNA-seq provides comprehensive data, identifying thousands of differentially expressed genes (DEGs) and enabling functional enrichment analyses [24,53]. Preliminary findings from RNA-seq studies in horses have revealed significant associations between emaciation and altered metabolic pathways, including oxidative phosphorylation and ATP synthesis [17]. These results underscore RNA-seq’s ability to uncover novel molecular targets and pathways, which are critical for understanding the complex metabolic shifts occurring in starved horses. Furthermore, RNA-seq’s capacity to analyze diverse tissues, such as fat, liver, and gastrointestinal tract, positions it as a superior method for advancing equine metabolic research [25,36,53].

RT-qPCR, traditionally employed in equine gene expression studies, has limitations that constrain its utility. While it offers precision in quantifying specific gene expression levels, its dependency on preselected targets and susceptibility to contamination and amplification errors restricts its scope [48,49,52]. In studies focusing on metabolic genes in starved horses, RT-qPCR has shown inconsistent results, partly due to challenges in primer design and tissue-specific variability [105]. Additionally, differences in euthanasia methods and post-mortem tissue handling may affect gene expression measurements, further complicating interpretations [42,47]. Despite these challenges, RT-qPCR remains a valuable tool for validating RNA-seq findings and for targeted investigations of specific genes of interest [48].

There remain significant gaps in the research on metabolic alterations in starved horses. Most existing studies rely on methods that either lack sensitivity or fail to provide a holistic view of metabolic changes [70,84]. For instance, while RNA-seq has demonstrated its potential, its application in equine studies has been limited, particularly in tissues beyond skeletal muscle [17]. Similarly, the selection of reference genes for RT-qPCR often lacks standardization, leading to variability in results [50]. Moreover, there is limited exploration of how different refeeding strategies impact the recovery of metabolic functions in starved horses [19,82]. Addressing these gaps requires integrating advanced omics technologies like RNA-seq with robust experimental designs and exploring broader tissue types to develop a more comprehensive understanding of equine metabolic resilience and recovery.

Characterizing metabolic consequences of starvation in horses has progressed, yet substantial gaps persist in both methodology and research scope. RNA-seq offers a promising tool for uncovering new insights, but limitations inherent to traditional techniques, such as RT-qPCR must also be addressed [24,52]. Expanding research to include diverse tissues and refining experimental protocols will be essential for advancing equine metabolic science, ultimately improving welfare and refeeding strategies for starved horses [18,23,25].

## 6. Implications and Future Directions

The findings have significant implications for the equine industry, particularly in developing evidence-based guidelines for refeeding protocols. Insights from related fields, such as animal nutrition and veterinary pathology, can offer valuable lessons for managing emaciated horses. For example, studies in animal nutrition emphasize the importance of gradual dietary adjustments to prevent metabolic disturbances, with direct application to refeeding protocols for starved horses. Similarly, veterinary pathology research on systemic inflammation and tissue recovery provides a framework for understanding the physiological challenges during recovery. By integrating these interdisciplinary insights, minimizing complications and improving welfare outcomes for emaciated horses is achievable.

Emerging trends in the field include the use of metabolomics and transcriptomics to provide a holistic view of physiological changes during starvation and recovery. Studies have demonstrated the potential of newer approaches, such as RNA-seq and metabolomics, to uncover novel biomarkers and therapeutic targets [58,106]. Recent advancements in omics technologies, such as proteomics and lipidomics, further allow for a deeper understanding of cellular responses to nutrient deprivation [18]. Additionally, the integration of machine learning approaches into the analysis of large datasets is enabling predictive modeling of recovery outcomes in starved animals. These tools can identify key predictors of health recovery and optimize intervention strategies, bridging the gap between molecular findings and clinical applications [107]. Future research should focus on longitudinal studies that integrate molecular, physiological, and clinical data to better understand the recovery process in horses.

## 7. Conclusions

The review of current literature underscores the complex metabolic alterations that occur in horses due to prolonged starvation and highlights the physiological, molecular, and methodological challenges involved in studying and rehabilitating emaciated equines. Starvation induces a significant reorganization of energy metabolism, characterized by reduced glucose utilization, increased reliance on gluconeogenesis, muscle protein breakdown, and enhanced lipid oxidation. Widespread alterations in gene expression across carbohydrate, protein, and lipid pathways reflect these metabolic changes. As starvation progresses, the body prioritizes energy conservation and the maintenance of essential organ functions, often at the cost of muscle mass and metabolic flexibility. Understanding these physiological changes at the molecular level is essential for developing effective, evidence-based refeeding protocols and improving clinical outcomes for starved horses.

Compounding the complexity of studying metabolic gene expression in starved horses is the methodological impact of euthanasia and post-mortem tissue collection. The use of sodium barbiturate, while a humane standard in veterinary care, influences transcriptional activity, including inflammatory and metabolic responses, which can confound downstream RNA analyses. Variability in euthanasia protocols, drug dosages, and tissue collection timelines can obscure the biological differences researchers aim to measure, particularly when comparing animals in distinct physiological states. Ensuring consistency in these procedures, and considering alternative euthanasia methods where appropriate, is vital for producing reliable transcriptomic data. These methodological considerations are especially important as molecular tools like RNA-seq and quantitative PCR become increasingly central to equine metabolic research.

The growing application of RNA-seq and RT-qPCR in equine metabolic research holds great promise for identifying molecular biomarkers of starvation and guiding more effective refeeding protocols. However, to maximize the translational value of these technologies, future research must address current gaps, including the lack of breed-specific data, the influence of age and body condition, and the need for standardized reference genes in equine studies. Integration of transcriptomic findings with clinical outcomes will enable the development of precision-based nutritional rehabilitation strategies, improving survival rates and long-term health outcomes for starved horses. Ultimately, expanding our understanding of equine metabolic gene expression will not only enhance animal welfare but also provide critical tools for veterinarians, caretakers, and policymakers in addressing the ongoing crisis of equine neglect and overpopulation.

## Figures and Tables

**Table 1:** Specific genes associated with CHO metabolism and starvation in equine tissues.

Gene	Function Related to Starvation and CHO Metabolism	Citation
SLC2A4	Downregulated during starvation, reducing insulin-stimulated glucose uptake in skeletal muscle and adipose tissue, conserving glucose for essential tissues.	[33,85,88]
HK2	Decreased expression under energy deprivation, limiting glycolytic flux and preserving glucose for gluconeogenesis.	[34,91]
PFKM	Key glycolytic enzyme that controls the commitment step of glucose breakdown; its activity can be modulated during starvation as glycolysis slows and glucose is conserved for essential tissues.	[92,93]
PDK4	Induced during fasting to inhibit pyruvate dehydrogenase, suppressing glucose oxidation and promoting fatty acid use.	[87]
INSR	Reduced signaling in prolonged starvation decreases peripheral glucose uptake.	[81,94]
GYS1	Suppressed to prevent glycogen synthesis, preserving glucose.	[89,95]
AMPK	Activated in low-ATP states during starvation, shifting metabolism toward fat oxidation.	[68,81]
SLC2A2	Alters hepatic glucose transport to favor endogenous production.	[73,89]

**Table 2:** Specific genes associated with protein metabolism and starvation in equine tissues.

Gene	Function	Citation
MSTN	Elevated during starvation to suppress muscle growth and promote protein catabolism.	[66,71]
IGF1	Downregulated in starvation, reducing anabolic signaling and muscle protein synthesis.	[94,96,97]
FBXO32	Upregulated during energy deficiency, tagging proteins for degradation.	[38]
TRIM63	Promotes proteasomal degradation of myofibrillar proteins in starvation.	[38]
MTOR	Suppressed to downregulate protein synthesis and activate autophagy.	[81,98]
SLC1A5	Facilitates glutamine uptake to fuel gluconeogenesis.	[41]
BCAT2	Catalyzes first step in BCAA catabolism.	[81,99]

**Table 3:** Specific genes associated with lipid metabolism and starvation in equine tissues.

Gene	Function	Citation
CPT1B	Transports long-chain fatty acids into mitochondria for β-oxidation.	[39]
ACADM	Oxidizes medium-chain fatty acids.	[40,41,75]
ACADL	Oxidizes long-chain fatty acids.	[75,100]
PPARGC1A	Induced to promote mitochondrial biogenesis and fat utilization.	[64,101]
FABP4	Facilitates intracellular transport of mobilized fatty acids.	[102,103]
LPL	Hydrolyzes triglycerides to free fatty acids.	[72,103]
PPARA	Activates fatty acid uptake and oxidation genes.	[75,81]
SCD1	Converts saturated to monounsaturated fats.	[81,104]
PLIN1	Regulates lipid droplet breakdown.	[81]

## Abbreviations

The following abbreviations are used in this manuscript:

RNA-seq	RNA Sequencing
RT-qPCR	Real Time Quantitative Polymerase Chain Reaction
BCS	Body Condition Score
CHO	Carbohydrate
